# Gene Expression Prediction by Soft Integration and the Elastic Net—Best Performance of the DREAM3 Gene Expression Challenge

**DOI:** 10.1371/journal.pone.0009134

**Published:** 2010-02-16

**Authors:** Mika Gustafsson, Michael Hörnquist

**Affiliations:** Department of Science and Technology, Linköping University, Norrköping, Sweden; Center for Genomic Regulation, Spain

## Abstract

**Background:**

To predict gene expressions is an important endeavour within computational systems biology. It can both be a way to explore how drugs affect the system, as well as providing a framework for finding which genes are interrelated in a certain process. A practical problem, however, is how to assess and discriminate among the various algorithms which have been developed for this purpose. Therefore, the DREAM project invited the year 2008 to a challenge for predicting gene expression values, and here we present the algorithm with best performance.

**Methodology/Principal Findings:**

We develop an algorithm by exploring various regression schemes with different model selection procedures. It turns out that the most effective scheme is based on least squares, with a penalty term of a recently developed form called the “elastic net”. Key components in the algorithm are the integration of expression data from other experimental conditions than those presented for the challenge and the utilization of transcription factor binding data for guiding the inference process towards known interactions. Of importance is also a cross-validation procedure where each form of external data is used only to the extent it increases the expected performance.

**Conclusions/Significance:**

Our algorithm proves both the possibility to extract information from large-scale expression data concerning prediction of gene levels, as well as the benefits of integrating different data sources for improving the inference. We believe the former is an important message to those still hesitating on the possibilities for computational approaches, while the latter is part of an important way forward for the future development of the field of computational systems biology.

## Introduction

The massive growth of high throughput data within molecular biology during the last decade has sparked an interest in systems biology and generated a great variety of suggestions on how to infer knowledge from these data sets. That is, whether the data belong to the genomics, transcriptomics, proteomics or metabolomics domain, they still need to be structured before one can learn anything from them. Here, networks have proved to be a unifying language for different biological systems involving, genes, proteins, metabolites and also small molecules. These networks, defined by protein-protein, protein-to-gene, metabolic interactions etc., determine cellular responses to input signals and govern cellular dynamics [1]. Still, though, the relative benefits of the proposed structuring methods are unclear, in part since researchers mainly publish positive merits benchmarked on their own data sets. Therefore, it was very welcome when the DREAM (Dialogue on Reverse Engineering Assessment and Methods) project was presented in 2006 during a conference [2]. Here at last, researchers had the opportunity to compare their algorithms in an objective manner. The first challenge, called DREAM2, was held between July and October 2007, and the outcome was presented both in a dedicated conference and in a special issue of the Annals of the New York Academy of Sciences [3]. The initiative was appreciated by the community, and in June 2008 the DREAM3 challenges were presented [4]. Compared with DREAM2, some of these challenges were turned to issues where the predictions could be directly measured, and were in this sense more realistic. Of special interest for the present authors was the challenge of predicting rankings of expression values for 50 genes in one time-series, where a compendium of 9335 probes for 32 expression profiles, divided into four time-series corresponding to various mutants, of yeast, *Saccharomyces cerevisiae*, were presented (the values for the searched genes were of course removed for the time-series of interest). One was also allowed to utilize any public data available.

Integration of data, which this challenge implicitly called upon, has been the subject of much attention recently; see for example the review by Hecker et al. [5]. There are several rationales for merging data when analyzing the outcome of high-throughput experiments. First and foremost is the fact that the systems and networks one infers often have so many units/nodes that the problem is not well-posed, for any mathematical model, due to lack of data [6], [7] (unless one introduces further constraints, such as sparseness). This is especially true when the measurements have been genome-wide, which means that they comprise data from thousands of units/genes, while the number of measurements for one condition seldom exceeds a few hundred. Another rationale is the quality of the data, which often is low. Therefore, it is of importance to strengthen the quality of the inference process by guiding it as much as possible with data corresponding to various angles of approach. In this article we present our contribution to the DREAM challenge, both describing which data we integrated and how the inference algorithm was developed. We also analyze our result, something which could be done first after the submission period was over and the observed values were released. The paper starts with a survey of the specific challenge for the DREAM competition, followed in the next section by the results we obtained. In this result section, we also compare the performance of our algorithm with others participating in the challenge. Thereafter, we have a discussion on what can be learnt from this exercise and suggest some lines of future research. In the methods section, we give a description of how we developed our algorithm; especially we describe in detail both how we integrate more expression data from other conditions and utilize information on TF (transcription factor) bindings.

### The Gene Expression Prediction Challenge of DREAM3

The challenge for predicting gene expression provided by the DREAM project is of great importance to explore the benefits and bottlenecks of the state-of-the-art algorithms in a fair competition. It represents a solution to the non-trivial problem of designing relevant challenges which at same time addresses biological and computational interesting problems. From the DREAM web-site [wiki.c2b2.columbia.edu/dream/index.php/The_DREAM_Project, accessed October 10, 2008] we quote for the gene expression prediction challenge within DREAM3:

Gene expression time course data is provided for four different strains of yeast (*S. Cerevisiae*), after perturbation of the cells. The challenge is to predict the rank order of induction/repression of a small subset of genes (the “prediction targets” in one of the four strains, given complete data for three of the strains, and data for all genes except the prediction targets in the other strain. Predictors are also allowed to use any information that is in the public domain but are expected to be forthcoming about what information was used.

**Background.** GAT1, GCN4, and LEU3 are yeast transcription factors. Each of these transcription factors has something to do with controlling genes involved in nitrogen or amino acid metabolism. The genes are not essential because strains that have perfect deletions of any of these genes are viable. In this challenge, we provide gene expression data from four strains: (i) a strain that is wild-type for all three transcription factors (wt, or parental), (ii) a strain that is identical to the parental strain except that it has a deletion of the GAT1 gene (gat1Δ), (iii) a strain that is identical to the parental strain except that it has a deletion of the GCN4 gene (gcn4Δ), and (iv) a strain that is identical to the parental strain except that it has a deletion of the LEU3 gene (leu3Δ).

Expression levels were assayed separately in all four strains following the addition of 3-aminotriazole (3AT). 3AT is an inhibitor of an enzyme in the histidine biosynthesis pathway and, in the appropriate media (which is the case in these experiments) inhibition of the histidine biosynthetic pathway has the effect of starving the cells for this essential amino acid.

Data from eight time points was obtained from 0 to 120 minutes. Time t = 0 means the absence of 3AT.

**The challenge.** Predict, for a set of 50 genes, the expression levels in the gat1Δ strain in the absence of 3-aminotriazole (t = 0) and at 7 time points (t = 10, 20, 30, 45, 60, 90 and 120 minutes) following the addition of 3AT. Absolute expression levels are not required or desired; instead, the fifty genes should be ranked according to relative induction or repression relative to the expression levels observed in the wild-type parental strain in the absence of 3AT.

This challenge is biologically relevant, and the fact a gold standard exists but is hidden makes the challenge objective and fair. Further, the probe names were given, which allows for data integration of publicly available experiments and a priori knowledge, making the challenge even more realistic in describing a situation which can occur in one's laboratory. However, the problem is somewhat different from the normal setting in systems biology where the aim is not only to predict future experiments but also to obtain interpretable models from which we can gain an increased biological understanding [8]–[10]. The data for this DREAM challenge was kindly delivered by Neil Clarke and co-workers, a fact which was revealed first after the submission period for predictions had closed. We will henceforth refer to this data as the “DREAM data”.

## Results

The goal of the challenge of DREAM was to predict the order of the chosen 50 genes within the gat1Δ strain for the eight time points at which they were measured. All details about the algorithm we utilized and how it was developed can be found in the Methods section. When the gold standard was revealed, it turned out that the mean correlation we obtained was 0.563, and that we, together with a prediction submitted by J. Ruan [11] who obtained a mean correlation of 0.558, had performed substantially better than the other participants.

Comparing with the training results of Tables 1, 2, 3 from the Methods section, we also calculate the results when we apply our algorithm to the gat1Δ strain in its premature states:

- Table 1. Using only the expression values for obtaining a perfect fit and a L2-minimization of the coefficients, we get a rank correlation with the gold standard of 0.535. Actually, the highest value here is obtained for the case when both values and rates are included, and a L1-minimization of the coefficients is performed; in this case the rank correlation is 0.609. These numbers should be compared with Table 1, where we can see the correspondences 0.712 and 0.663, respectively, for these two methods.
- Table 2. Combining least squares with the elastic net for the gat1Δ strain gives a rank correlation of 0.620, while RLAD results in 0.320. Compare this with Table 2, which has corresponding values of 0.794 and 0.681.
- Table 3. The prediction for the gat1Δ strain has a correlation with the gold standard of 0.623 when more expression data are included. When we also add prior knowledge of TF-bindings, the correlation increases further to 0.624. These correlations correspond to the values 0.856 and 0.857 from Table 3.

**Table 1:** Spearman rank correlations for predictions obtained by perfect fits and minimization of L1- and L2-norms.

		Training strains			
Norm	Data	wt	gcn4Δ	leu3Δ	Overall
L1	values	0.616	0.632	0.686	0.670
L2	values	0.587	0.747	0.699	0.712
L1	values, rates	0.647	0.604	0.662	0.663
L2	values, rates	0.587	0.747	0.699	0.712
L1	rates	0.244	0.260	0.434	0.360
L2	rates	0.448	0.542	0.611	0.570

The correlations are based on cross-validations, where the last column stands for an overall calculation based on 24 ranking lists. The minimization with respect to an L2-norm has the best performance, both for including only expression values and for including both expression values and rates. Following the principle of including as little as possible, we discard the rates.

**Table 2:** Spearman rank correlations based on two different inference schemes.

	Training strains			
Algorithm	wt	gcn4Δ	leu3Δ	Overall
RLAD	0.486	0.799	0.684	0.681
LS, Elastic net	0.687	0.828	0.764	0.794

The correlations are based on cross-validations, where the last column stands for an overall calculation based on 24 ranking lists. A minimization of least squares, combined with a penalty term of the form of the elastic net, gives the best performance.

**Table 3:** Spearman rank correlations after soft integration of other data sets.

External data		Training strains			
Expression	TF-binding	wt	gcn4Δ	leu3Δ	Overall
x		0.793	0.881	0.789	0.856
x	x	0.793	0.880	0.791	0.857

The expression data are obtained from the Rosetta Inpharmatics and ncbi omnibus, and integrated into the inference process by more terms in the objective function. The TF-binding data come from Yeastract and form priors for the penalty term, making it more probable that genes which are co-regulated should act as predictors for each other. Both data sets are only included to the extent the cross-validation procedure allows.

An observation here is that the submitted prediction for the gat1Δ strain correlates less well with the gold standard than each of the series explored during the development of the algorithm. Neil Clarke points out in his referee report (published on-line accompanying this article) that he picked some of the genes to be predicted because of their surprising or non-trivial expression pattern the gat1Δ strain. This fact, combined with a general observation that cross-validation often underestimates the error [12], makes this result less surprising. Nevertheless, each step we take in the progression of the algorithm increases its performance, and as mentioned in the list above, we have the sequence of rank correlations as 0.535, 0.620, 0.623 and 0.624. The reason for these numbers being lower than the ones from the training of the algorithm is presently unclear to us.

**Figure 1:**
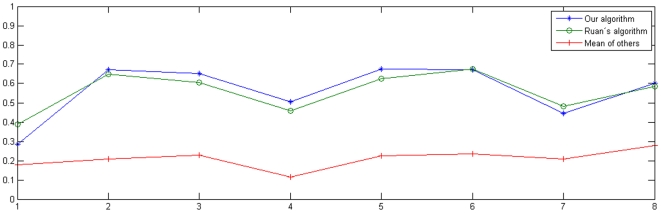
Spearman rank correlation for each time point. The correlations are all with respect to the gold standard. The upper blue curve (stars) is our result; the green curve slightly below (rings) belongs to Ruan [11], while the lower red curve (plus-signs) is the mean of all other participants. The connecting lines are only guides for the eye. Note how the rankings for some time points obviously are harder to predict than others, and that the results are clearly co-varying.

Considering the result for each time point, Figure 1, we can see a huge variation in how well we, and the other groups, succeeded. The upper blue curve (stars) shows the correlations we obtained for each time point, while the slightly lower green curve (circles) is for Ruan. The red curve with plus-signs is the mean of all other participants. We can see how some time points obviously are harder than others to predict. For example, for 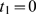 we obtained a correlation of only 0.285 but for 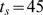 it is 0.675. Interestingly, the expression values which are harder to predict seem to be harder for all proposed methods, not only for our, and the curves co-vary. Especially, we can at 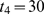 see how all algorithms make worse predictions than in nearby time points. Indeed, our result and the Ruan-result are correlated with a correlation coefficient of 0.957. The reason for this needs more research to find out.

**Figure 2:**
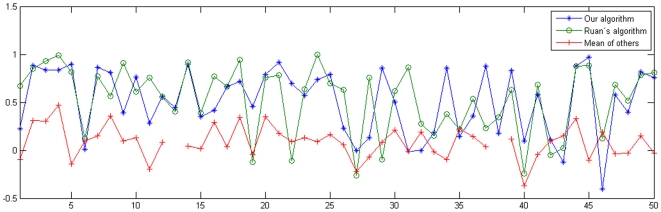
Spearman rank correlation for each gene. The correlations are all with respect to the gold standard. The upper blue curve (stars) is our result; the green curve slightly below (rings) belongs to Ruan [11], while the lower red curve (plus-signs) is the mean of all other participants. The connecting lines are only guides for the eye. Most of the times, these results co-vary, but occasionally they have even different signs. The two breaks of the mean curve (gene numbers 13 and 38) come from some non-valid numbers in the presented results.

If we instead consider the obtained rank correlations with the gold standard per gene, instead of per time point, we get the result in Figure 2. Also here, not surprisingly, the results by us and Ruan co-vary, but this time with the smaller correlation coefficient of 0.521. The variation this time is also considerably larger, where the highest rank correlation we obtain is 0.97 and the lowest is 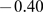. That is, for some genes we obtain orderings which are worse than lists picked by random. This is interesting, since it means there are a lot of improvements to be made. See, however, the discussion in [11] about the possible inappropriateness of using the Spearman rank correlation for the time-profile correlations the way it is done here, resulting in too pessimistic estimates of the performance of the algorithms. The reason is that the rank-transformation was performed for each time point, making comparisons between different times problematic. That is, this might introduce errors in estimating the time-profile accuracy. However, we refrain from any recalculations since then all submissions should be reconsidered.

## Discussion

The importance of challenges as DREAM lies to a large extent in its objectiveness. When an inference algorithm comes from the same laboratory as the one which has performed the assessment experiments, sometimes even in the same article, it is likely the algorithm has been tuned to fit with the expected outcome. This is most probably often over fitting, and decreases then its performance for other data sets. Also, the value of this procedure as an assessment is questionable, since the testing of only a few of the predictions of the algorithm has a clear anecdotic flavor, especially when the researcher can choose by him- or herself which parts should be presented. As a contrast, the DREAM challenges provide the community with workbenches where all are welcome to submit the predictions of their algorithms, and thereby getting the opportunity to assess and compare them with the performance of others. No one knows the gold standard beforehand, and even if the evaluation data is limited, it is well defined but still no fine tuning can be carried out. This makes a huge difference compared with the case mentioned above, when the same laboratory both performs the experiments and present inference algorithms with alleged generalizability.

However, this appreciated objectiveness and fairness of DREAM holds of course true only as long as the gold standard is hidden. As soon as it is revealed, one can start improving one's algorithm to better fit the expected outcome, but at the same time taking the risk of exposing it to over fitting. Any “improvements” at this stage must be very well motivated in order to make any sense at all. For example, for our algorithm, we could consider the possibilities to use local fitting parameters instead of a global one for the prior, or to further prune the model by choosing parameter values not at the cross-validation minimum, but one standard deviation below, etc. Due to the above mentioned reasons, we refrain from such actions, though, and instead look forward to the next round of DREAM.

The algorithm here presented represents one efficient way of predicting rankings of expression values. A key component in the development of the algorithm has been the inclusion of results of measurements not directly associated with the experimental condition for which the expression values should be predicted. Whether this inclusion has been for more expression data or for prior knowledge of TF-DNA bindings, a cross-validation scheme has helped us not to rely more on these measurements than the original data allow. This is denoted as “soft integration” and forms a cornerstone of our work. The success of the algorithm clearly shows that prediction of expression levels is a possible task, even when the number of genes in the system exceeds the number of experiments 100-fold.

Surprisingly, the inclusion of a priori knowledge of TF-DNA bindings did not improve the performance of the algorithm substantially. The reason for this needs more research to find out, since the quality of this kind of data is generally believed to be reasonably high. A hypothesis is that our choice to have just one global parameter 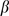 for tuning the impact for all genes was too restrictive.

Interestingly, the second best performance algorithm, by J. Ruan, is based on a very different thinking with respect to data integration. There, only the data provided by DREAM is utilized, and their algorithm is based on profile similarities measured by Euclidean distances and predictions from *k*-nearest neighbors, KNN [11]. Nevertheless, the performance of that algorithm was only on average slightly less satisfying than the performance of ours. Indeed, in not a few instances it even performed better than ours, and in [11] an even stronger version is presented, although it was presented first *after* the gold standard was revealed. When we consider the similarities per experiment (Fig. 1) or per gene (Fig. 2), we can see how our two algorithms seem to function equally well, with the exception of some genes (for example, number 19, 22, 29 and 46, as shown in Fig. 2).

The reason for the success, and failure, for both of these two philosophies for prediction of gene expression needs further research to find out. Especially, the cases where one algorithm is successful and the other is not, deserve extra attention. As a final remark, we stress again how the integration of data, which is important for our algorithm, did not at all appear in the Ruan-algorithm, but still the results are similar. That is, a simple method as KNN can still be as effective as a more sophisticated algorithm where TF-DNA bindings etc. are taken into account. This means there is probably a lot of improvements possible, which is a challenge for the future development of computational systems biology.

## Methods

### Modelling Assumptions

The quest for modelling gene networks has taken many different forms during the last decade [5], [9], [10], [13]. One of the major modelling frameworks is provided by ODEs, ordinary differential equations, which is the form we exploit here for the development of our algorithm.

An often utilized approach for large-scale modelling of gene regulatory networks is to only consider the transcripts, and thereby letting all interactions be projected onto the space of genes only [14]. By this, one obtains a gene-to-gene network, sometimes referred to as “‘influential’ gene regulatory network” [5]. Further, the amount of data available makes most models except linear ones behind reach, and one therefore assumes a linear relation between the expression rates and the expression levels [15]–[17] (or some non-linear transformation of them, the equations still being linear, though [18]). The rationale for this assumption of linearity is normally expressed as a belief in the system being close to some equilibrium or working point, and thus an expansion keeping only the linear terms would be appropriate. The basic dynamical equation then looks like
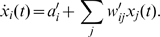
(1)Here 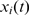 denotes the expression level of gene 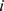 at time 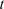, and the dot on the left hand side denotes its time-derivative. The coefficients 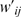 and the 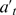 elements are to be inferred and describe the gene regulatory system on a gene-to-gene level. The interactions, i.e., the non-zero 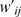, can both correspond to (semi)-direct interaction, for instance when 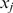 denotes the RNA-level of a transcription factor which binds to the promoter of gene 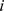, and indirect interactions, for instance when gene 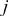 stands for an inhibitor of a transcription factor which in its turn regulate gene 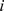. Note that the coefficient 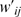 can be both positive, describing activation, and negative, describing inhibition. Since this is a dynamical equation, it can be used for studying the time-development of the model, including aspects as stability and flexibility [19].

Here, however, we are not primarily interested in the dynamics or in the derivatives of the 50 genes in the gat1Δ strain which were removed from the data file, but in the prediction of their expression levels. By denoting this set of 50 genes as 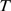, we reformulate and generalize Eq. (1) to
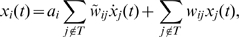
(2)for all 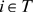.

### Model Selection

In order to obtain a ranking list based on the expressions of the 50 genes in 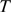, we must predict the values of 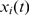 for all 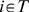 from (2). For this, we need explicit values of 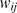 and 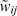. Given the values of 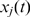 and 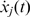 for all 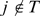 and 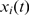 for all 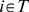, this can be formulated as a minimization problem with the objective function
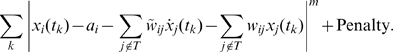
(3)We will here stick to the cases where 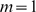, least absolute deviations (LAD), and 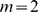, least squares (LS), respectively. The penalty term is utilized for discriminating among models, and is to be determined later. For the moment, it is set to zero. The experiments are assumed to have been performed at time 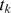, where we for ease of notation let the index 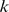 run over all three complete time-series (the gat1Δ-series, i.e., the series which should be predicted, is never utilized for the inference, since it is from this series we determine the output which is submitted for the challenge – hence there is a risk of over fitting if we utilize it twice).

The DREAM data are measured by Affymetrix chips of 9335 probes, and obtained from two biological and two technical replicates. We map the probe-names onto unique gene names, which leave 7804 units, where we use mean values when more than one probe corresponded to one gene. Furthermore, we approximate the derivatives from central differences, except at the end points of each series. At time 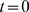 we set the derivative to zero, since it was right before the addition of 3AT, and at the final time 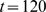 we approximate the derivatives from backward differences.

Throughout the article, we utilize cross-validation (CV) to discriminate among models. We hold one of the three time-series provided by DREAM out from the inference, and utilize the other two, and occasionally also other data sets, for finding the searched parameters. We then use data from the left out strain to predict the expression values of the 50 searched genes for each time point in the this series, rank them according to the predicted levels such that the highest expressed gene obtain rank number one, second highest rank number two, etc., and calculate the Spearman rank correlation with the observed ranking of the same series. This is repeated three times, holding each of the provided time-series out a time. We end up with 24 different ranking lists for the 50 genes in 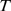, eight for each time-series. Henceforth, we will refer to this procedure simply as “cross-validation”. Note that this approach to only hold one of the given time-series out still holds also when external datasets are introduced.

Before we start exploring various versions of the penalty term in (3), we try to simplify the model (2). The strategy is to primarily work with the DREAM data, in order to reduce the model. When this first reduction is obtained, we will utilize also other publicly available data in order to further strengthen the predictive power of our mathematical model. This first model selection is performed among the models with perfect fits, i.e., the ones where the terms for the first sum of absolute values in (3) all are zero, making the exponent 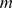 irrelevant, and we get an idea on what to include. This perfect fit is possible since for each gene value we should predict, that is, for each *i*, we have 15 509 free parameters to determine from 16 linear equations (one per time point). We explore the following three scenarios:

- Only expression values are included
- Both expression values and expression rates (derivatives) are included
- Only expression rates (derivatives) are included

By picking the solution with zero value for the objective function (without penalty term) and choosing the coefficients 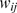 and 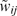 such that their L1-norm 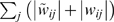 and L2-norm 
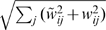
, respectively, are minimized, we obtain the values of the Spearman rank correlations presented in Table 1. The rank correlations are for each series comprising eight time points as well as for the full 24 lists simultaneously (referred to as “overall”) obtained by cross-validation. Note that this is not the same as the mean value of each time-series or time point.

We see that the highest values for the correlations are obtained when we only include the expression levels. Inclusion of the expression rates makes the result slightly worse, except for the least squares where the correlations are equal. However, with the same predictive power, we apply Occam's razor and prefer the simplest model. To only use the rates gives the least satisfying result of them all. Therefore, in the sequel, we choose to discard all derivative terms and determine the parameters according to
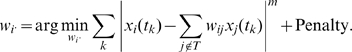
(4)Here we use the short-hand notation 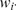 for 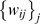 and drop the intercept term 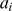 since we also centre the data. What remains is to determine the value of *m* and the explicit form and value of the penalty term.

By choosing the exponent 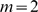 we turn the problem into Least Squares (LS), while 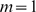 gives Least Absolute Deviations (LAD). Least squares are known to be sensitive for outliers, while LAD is not affected by such at all. However, the solutions obtained from LAD are unstable, in the sense that small variations in data might result in large variations of the inferred parameters, that is, least squares are a more stable inference method. Indeed, LAD can be compared with picking the median, which may be devastating when we are already short of data [20]. Both methods will here be explored.

The penalty term can take many different forms. A review for least squares of more classical forms as Mallow's Cp, Akaike Information Criterion (AIC), Bayesian Information Criterion (BIC), Minimum Description Length (MDL) etc in the context of gene networks can be found in [21]. We will here stick to forms which are computationally attractive, which is important since the size of the present problem makes methods based on exhaustive search practically impossible. We choose for least squares the form
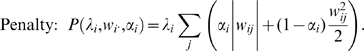
(5)This is known as the elastic net [22] and is a convex combination of the well established methods of ridge regression 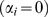 [23] and of the lasso 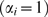, [24]. For LAD, least absolute deviations, only the “lasso-form”, i.e., 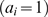, is of practical interest due to implementation considerations. This case is called regularized least absolute deviation (RLAD) [25].

This convex combination is a compromise between the two goals of a good solution, to have good predictive power and to be interpretable [5]. The L1 penalty in the lasso favours sparse solutions, i.e., it performs effectively a subset selection, but is greedy in the case of correlated predictors since it only picks the most correlated predictor. On the other hand, the L2 penalty in ridge regression keeps all predictors as non-zero with probability one, but is not greedy as the lasso. It has been argued that this compromise can overcome some of the limitations of the lasso and ridge regression, with the preservation of the benefits from each of the pure methods [22]. In Table 2 we investigate what kind of penalty is most predictive for our purpose. We investigate the elastic net for least squares 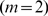 and RLAD for least absolute deviations 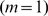 on the DREAM data set. The parameters 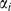 and 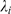 are obtained from an exhaustive grid search over the parameter space 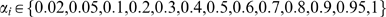 and 1000 equidistant values of 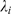 between zero and the upper limit (defined as the limit where the found solution coincides with the solution without penalty term). The parameters are eventually chosen with cross-validation. In practice, we utilize the R-package glmnet by Friedman et al. [26]. It turns out that the values of 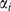 vary with 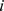 in the whole interval between zero and one, with a median of 0.75. That is, the solution is “lasso-like”, but still the maximum in-degree turns out to be 488 (with median 23.5) which is not possible for a pure lasso where the number of predictors cannot exceed the number of experiments.

We show in Table 2 the results of such an optimization. The entries are the Spearman rank order correlations obtained from cross-validation, i.e., they are the best measure of performance we have without adding any prior information. The general conclusion from Table 2 is that least squares regression combined with the elastic net is to prefer among the methods. From a correlation of 0.794 it is worthy to proceed with further improvements by integrating more data into the inference process. Worth noting is that in this process, the parameters 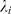 and 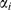 are not fixed, but will be recalculated by new cross-validation procedures.

### Data Integration

One way to improve the performance of the algorithm is to include more data. This is a challenging problem which is crucial for all kinds of large-scale inference problems [5]. Previous work has shown, however, that uncritical inclusion of more data can even decrease the performance of an algorithm [27]. For yeast, huge amounts of public data are available, which potentially can improve the quality of the inferred models. The main issue is to select and process only the relevant data sets. This must be done in a careful, supervised way to avoid over fitting, because validation data are on the other hand severely limited. The here presented framework introduces two main possibilities, integration of more expression data and introduction of prior knowledge of regulatory interactions.

#### More expression data

A straightforward way to include other types of expression sets is to extend the sum of squares in (4) over more data sets. We integrate two collections of expression sets, which reduce the number of possible genes to use as explanatory variables further; down to 4140 genes (since the numbers differ across the experiments, and we only utilize those genes for which we have measurements in all experiments). The final collection comprises:

- A set of total of 515 steady-state profiles from a collection of the gene knock-out experiments [28] and other heavy perturbations [29] from the Rosetta Inpharmatics
- A set of 256 time-series profiles, comprising a collection of time-series experiments downloaded from ncbi omnibus [30], [31] with GEO accessions GDS16, GDS18, GDS19, GDS20, GDS30, GDS33, GDS34, GDS36, GDS37, GDS38, GDS39, GDS104, GDS108, GDS109, GDS112, GDS113, GDS115, GDS124, GDS180, GDS283, GDS354, GDS608, GDS1013, GDS1752, GDS2267 and GDS2715

However, the experimental conditions can vary a lot, and most of them are probably distant from the conditions we actually are interested in. It is therefore likely that these profiles have less impact on the actual problem than the primary profiles presented for the actual problem. We therefore introduce an extra coefficient 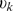 for each term in (4), which becomes
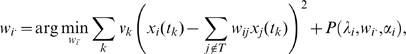
(6)These coefficients are chosen to be unity for the DREAM data, to set the scale, and we determine their values otherwise by the cross-validation procedure. In order not to have too many free parameters in the model, we pick one value for the 515 steady state profiles from Rosetta, 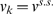 and another value for the 256 profiles from the time-series of ncbi omnibus, 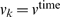. The result is shown in the first line of Table 3.

#### Prior knowledge of TF-DNA regulations

The TF-DNA binding data are taken from Yeastract [32], [33], and give clues on which genes are likely to act as regulators. To take this knowledge into account, we utilize a modified form of the penalty in (5). Now, we choose a different penalty for each predictor such that we increase the probability for a non-zero 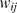 if gene *j* somehow, to be specified below, is co-regulated with gene *i*. By utilizing the cross-validation scheme, we integrate this prior knowledge in a soft way, i.e., we bias the search to known relations, but allow also the possibility of novel links. Explicitly, expression (5) is modified by the introduction of parameters 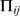, called priors, reflecting the likeliness of gene 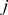 *not* to be co-regulated with gene *i*, to look like
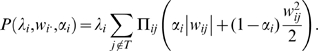
(7)That is, the prior 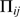 corresponds to our prior belief that there is *no* correspondence between gene *j* and gene *i*, since a high value of 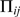 implies a high penalty, and thus there has to be a high correlation between the genes to include gene *j* as a predictor for gene *i*. We let the parameters 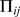 be between zero and unity, with no loss of generality, since it is only the relative difference which is of relevance, while 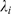 takes care of the global magnitude of the penalty.

**Figure 3:**
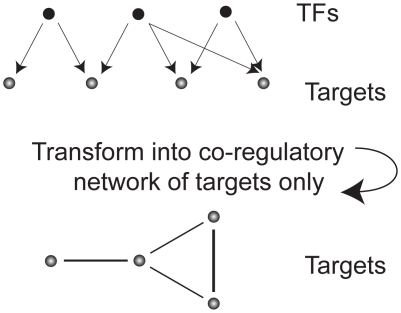
Schematic view of how to obtain the weighed shared fraction of TFs. We utilize as prior information that genes which are co-regulated are likely to be effective predictors of each other; the more TFs in common, the more likely to be co-regulated.

The reason why we focus on co-regulation rather than regulatory interactions is that the values of the inference are based on transcript levels, and TFs are known to be expressed on a low level. Also, their activity is often determined by phosphorylation and other effects rather than their amount [34], which makes the levels even more dubious. Therefore, we concentrate not on the TFs themselves but on genes which are controlled by the same TFs and thus might act as predictors for each others. The rationale can also be formulated such that if two genes are regulated by the same set of TFs they are likely to have large (anti-) correlation, thus the level for one of the genes should be a good predictor for the other, see Figure 3. The explicit calculation is carried out through the TF interaction matrices obtained from Yeastract [32], [33]. We set 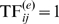 if there is documented experimental evidence for TF-*j* binding upstream of gene *i* and 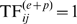 if there is either experimental evidence or putative evidence (or both) for such a binding. Otherwise, the elements are set to zero. From these TF interaction matrices, we calculate the weighted shared fraction of TFs between gene *j* and gene *i*, 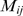, as
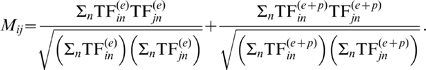
(8)Note that 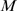 is a symmetric matrix, 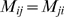, just as it should from its definition of a weighted shared fraction of TFs. The prior finally is chosen as:
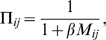
(9)where 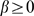 is a free global parameter to be determined later by cross-validation.

To summarize the discussion above, the objective function takes the form
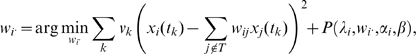
(10)where the penalty term is
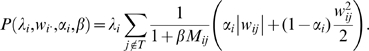
(11)Thus, the data integration leaves us with 207,103 parameters (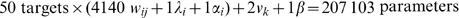) in total to determine. The global parameters result in the fractional value of 4142.06 parameters for each gene to predict. That is, the cross-validation procedure we utilize has to minimize expression (10) with a penalty term of the form presented in (11) and detailed in (8). We minimize it by a greedy stepwise procedure, again using the R-package glmnet [26].

Effectively, for each target gene 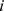, we start from the value of 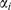 found without any prior and explore 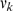 and 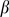 individually. For each sets of values for these parameters, we run glmnet with 1000 values of 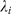 (described above) and perform local searches in 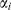 around its present value, changing it if the Spearman rank correlation increases. The parameters 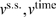 and 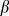 are introduced in the order they increase the performance of the algorithm, but when a value is determined, we do not change it again. It turns out they are introduced in the order 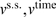 and 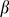, with the values 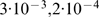 and 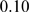, respectively. They are obtained by in an iteratively refinement process, where the initial searches for 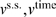 are in the range between zero and one, and values for 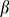 from the set 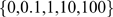. The refined search utilizes steps of the size 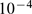 for 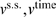 and 0.01 for 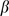. The result is shown in the second line of Table 3. We can see how this final step further increases the quality of the performance of the algorithm, although the increase this time is not as drastic as before. Worth noting is also how the Rosetta data contribute ten times more than the data from the ncbi omnibus. The rather low value of 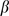 is also an indication that the positive contribution of known TF-DNA bindings is limited.

Finally, from a computational point of view, we remark that all implementations and calculations have been performed on an ordinary laptop in the languages R and Matlab. That is, the complexity of the problem is not worse than it can be handled in any laboratory.
